# Diversity and Bioprospection of Functional Proteins from Sea Anemone *Heteractis magnifica* Based on Multi-Omics Approach

**DOI:** 10.3390/md24080276

**Published:** 2026-08-07

**Authors:** Jiao Chen, Zhen Chen, Shibo Sun, Chang Lyu, Ming Li, Yun Song, Bingmiao Gao

**Affiliations:** School of Pharmaceutical Sciences, Hainan Medical University, Haikou 571199, China; hy0207109@muhn.edu.cn (J.C.); chenzhen@hainmc.edu.cn (Z.C.); sunshibo@hainmc.edu.cn (S.S.); 14783160355@163.com (C.L.); mingli@hainmc.edu.cn (M.L.)

**Keywords:** *Heteractis magnifica*, functional proteins, transcriptomic, proteomic, AlphaFold3 modeling, molecular docking

## Abstract

Sea anemone venom has attracted increasing attention in biomedical research due to its multifarious compounds with biological activities. Although the venom is predominantly made up of proteins, the diversity and complexity of these proteins remain poorly understood. In this work, the proteins derived from the tentacle and column of *Heteractis magnifica* were investigated by integrating transcriptomic and proteomic technologies. A total of 3573 protein sequences from transcriptome databases were identified and clustered into nine functional categories. We also performed proteomic analysis on the proteins identified in *H. magnifica*, and 339 proteins were found to be present in both datasets. Notably, a comprehensive analysis of six typical categories was implemented, and the representative proteins were explored in depth using multiple alignments, homology modeling and molecular docking. Meanwhile, a few low-copy but functionally intriguing proteins were discovered, highlighting the presence of unconventional components in sea anemone venom. This work provides the first holistic overview of the typical protein families and novel information on functional proteins from *H. magnifica*, contributing to a deeper understanding of sea anemone proteins and facilitating the discovery of potential proteins for marine drugs or biotechnological tools.

## 1. Introduction

Cnidarians, unlike venomous animals that possess dedicated venom glands, produce and deliver venom through their unique intracellular organelles—the nematocysts—which are primarily employed for prey capture, defense, and interspecific competition [1]. Among this phylum, sea anemones (Actiniaria) represent one of the most species-rich and ecologically prominent groups, accounting for a substantial proportion of cnidarian diversity and serving as key models for venom studies. Although nematocysts are distributed across various body regions, they are the most densely concentrated in the tentacle, which are the primary sites for venom deployment [2,3]. However, it is noteworthy that the basal disk, though primarily serving as an attachment structure, also harbors a certain number of nematocysts, suggesting a potential supplementary role in defense or localized interactions with the substrate. The venom of sea anemones is a multitudinous mixture of small molecules, peptides and proteins, which are fascinating for marine bioprospecting due to the potential for novel natural marine products to be found [4,5]. Among these compounds, peptide toxins constitute the best studied group with regard to diverse bioactivity and potential application for human diseases. For instance, Dalazatide, derived from well-known ShK peptide, has been verified in the inhibition of K_v_ 1.3 channels in human T lymphocytes, making it an excellent candidate for clinical therapeutics of autoimmune diseases [6]. However, only a few literatures focus on the proteins of sea anemones and their potential functions, since the protein is the largest component of sea anemone toxin. Herein, it is also worth paying attention to the diverse proteins of sea anemones.

In recent years, advances in multi-omics technologies—encompassing proteomics, transcriptomics, and genomics—have revolutionized the study of sea anemones. These approaches integrated with bioinformatics tools allow for high-throughput identification and characterization of venom components, uncovering hundreds of novel toxin-like molecules and associated pathways. Transcriptomic studies have identified numerous gene families linked to venom production, shedding light on the molecular machinery driving venom diversity [7,8]. Proteomic analyses, on the other hand, have revealed a repertoire of venom peptides involved in diverse biological processes. So far, there are nearly 1200 sea anemone species, yet only less than 4% of these species have been isolated and identified to investigate their venom peptides and proteins [4]. Of these, nearly 20 species have undergone transcriptomic studies, whereas only 6 have been examined using integrated multi-omics approaches, including *Heteractis magnifica*, *Stichodactyla haddoni*, *Anthopleura dowii*, *Bunodosoma caissarum*, *Entacmaea quadricolor*, and *Nematostella vectensis* [3,5,9,10,11]. Compared with a single-omics approach, the multi-omics approach could be more accurate, since the results derived from transcriptomic can further be validated by proteomics at protein level, avoiding false positive results.

The starlet sea anemone *H. magnifica* (Family Stichodactylidae: Genus Heteractis), is a species known to occur in the tropical regions of the Indian–Pacific Ocean. The specimens used in this study came from the South China Sea. The complexity and diversity of peptide toxins in *H. magnifica* had been firstly elucidated by our group [10], yet its protein toxins remain unexplored. Therefore, this paper aims to provide a deep insight into the structural and functional diversity of sea anemone proteins through multi-omics approaches. Compared with emphasis of previous studies on the peptides of sea anemones, this work provides the first holistic overview of the typical protein families, predicts their 3D structures and evaluates their potential activities through molecular docking. These findings deepen our understanding of sea anemone proteins and support the discovery of functional candidates for novel marine drugs.

## 2. Results

### 2.1. Putative Proteins Identified in the Transcriptomes of the Tentacle and Column of H. magnifica

The original sequencing data of *H. magnifica* were first reported by our group in a previous study, and subsequently deposited in the National Center for Biotechnology Information (Bioproject: PRJNA1023041) [10]. Briefly, the 41,408,576 raw reads from the tentacle and 44,188,792 raw reads from the column were generated by Illumina HiSeq. Using the Trinity software v2.15.1, after quality filtering, transcriptome assembly yielded 134,263 tentacle transcripts (avg. 675 bp, N50 = 1105 bp) and 94,024 column transcripts (avg. 721 bp, N50 = 1256 bp) from 207,530 paired reads from the tentacle and 148,422 paired reads from the column, respectively. Moreover, the functional annotations were also previously performed by our group against Non-Redundant Protein Sequence Database (Nr), Universal Protein Resource (UniProt), Kyoto Encyclopedia of Genes and Genomes (KEGG), Clusters of Orthologous Groups for Eukaryotic Complete Genomes (KOG) and Gene Ontology (GO) databases. As a result, a total of 44,831 tentacle genes and 29,174 column genes were functionally annotated from the 134,263 unigenes from the tentacle and 94,024 unigenes from the column, respectively.

**Figure 1:**
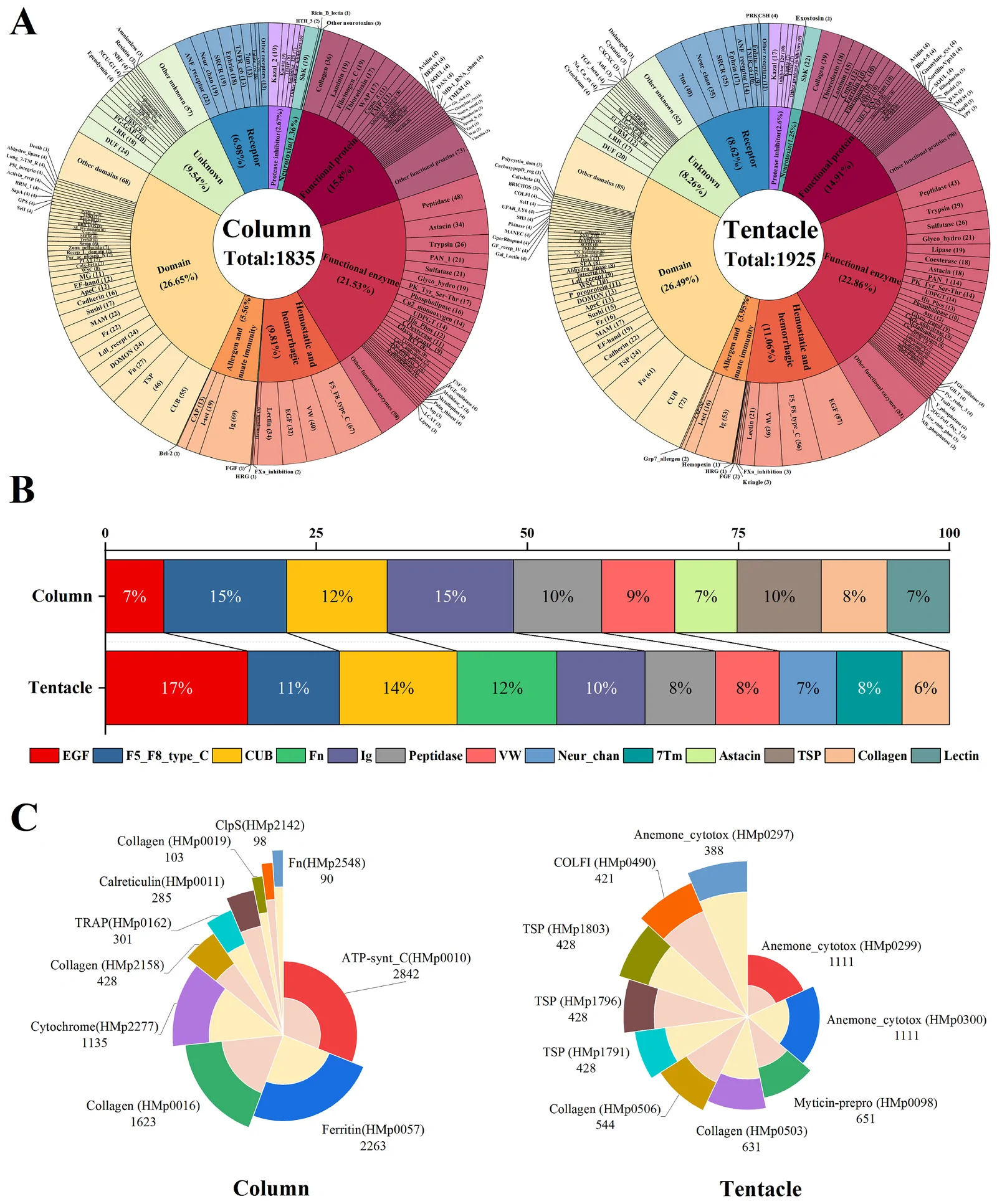
Summary of identified proteins from the transcriptomes of *H. magnifica*. (**A**) The classification of putative protein sequences from the column and the tentacle. Inner circle: biological function and overall percentage of each category. Outer circle: gene families within each biological function category. (**B**) Stacked bar graph showing the proportions of the top 10 gene families from the column and the tentacle. (**C**) Ten transcripts of proteins with the highest levels of transcription and their corresponding families from the *H. magnifica* transcriptomes.

In this study, the assembled transcripts were manually also searched against UniProt and InterPro databases, aiming to identify functional proteins that *H. magnifica* produces and their potential value in biological applications. A total of 3573 protein sequences were identified (Supplementary Figure S1), of which 1738 were exclusively identified from the tentacle (Supplementary Table S1), 1648 from the column (Supplementary Table S2), and 187 in both tentacle and column (Supplementary Table S3). Of these, 1925 protein sequences from the tentacle were identified and divided into 144 known families, while 1835 protein sequences from the column were identified and classified into 142 groups (Figure 1A). According to the biological function, all these families could be further categorized into nine functional categories, including mixed domain, functional enzyme, functional protein, hemostatic and hemorrhagic toxin, allergens and innate immunity, protease inhibitor, neurotoxin, receptor and unknown functional protein. Overall, the tentacle possessed slightly more proteins (1925 vs. 1835), reflecting higher molecular complexity. Functionally, tentacle showed higher proportions of hemostatic/hemorrhagic proteins (11.06 vs. 9.81%), receptors (8.62 vs. 6.98%), and enzyme (22.86 vs. 21.53), aligning with their frontline roles in environmental sensing, prey capture, and injury repair. Conversely, the column had a higher proportion of allergen-related protein (5.67% vs. 4.36%) and functional protein (15.80% vs. 14.91%), which were predominantly involved in extracellular matrix construction and immune recognition, further confirming the core role of the column in tissue stabilization, substrate attachment, and basal defense. As shown in Figure 1B, the proportions of the top 10 most abundant gene families in the total recovered protein arsenal of each focal tissue were reported. Transcripts identified as immunoglobulin (Ig, 15%, 69), coagulation factor V and VIII (F5_F8_type_C, 15%, 67), CUB domain (12%, 55), peptidase (10%, 48) and thrombospondin domain (TSP, 10%, 46), were the most abundant gene families in the column, while epidermal growth factor (EGF, 17%, 87), CUB domain (14%, 72), fibronectin (Fn, 12%, 61), F5_F8_type_C (11%, 56) and Ig (10%, 53) were the most abundant gene families in the tentacle. Despite some differences in family composition between the column and the tentacle, both tissues were rich in hemolytic and coagulation-related families including Ig, F5_F8_type_C, EGF, Fn and TSP. Transcripts per million (TPM) values were employed to quantify the transcript levels of genes in different tissues (Figure 1C). The results revealed that HMp0010 assigned to ATP synthase subunit C domain (ATP-synt_C) exhibited the highest transcript levels in column tissue, immediately followed by ferritin, collagen, and cytochrome protein, which collectively point to robust energy metabolism, iron storage/detoxification, and structural support. Whereas, HMp0299 and HMp0300, which belong to anemone cytotox protein, were the most prominent in the tentacle region, followed by myticin-prepro antimicrobial protein, and TSP. The high expression of these signature proteins directly underscores the tentacle’s physiological role as a “biochemical arsenal” for predation and chemical defense.

### 2.2. Putative Proteins Identified in the Proteomes of the Tentacle and Column of H. magnifica

**Figure 2:**
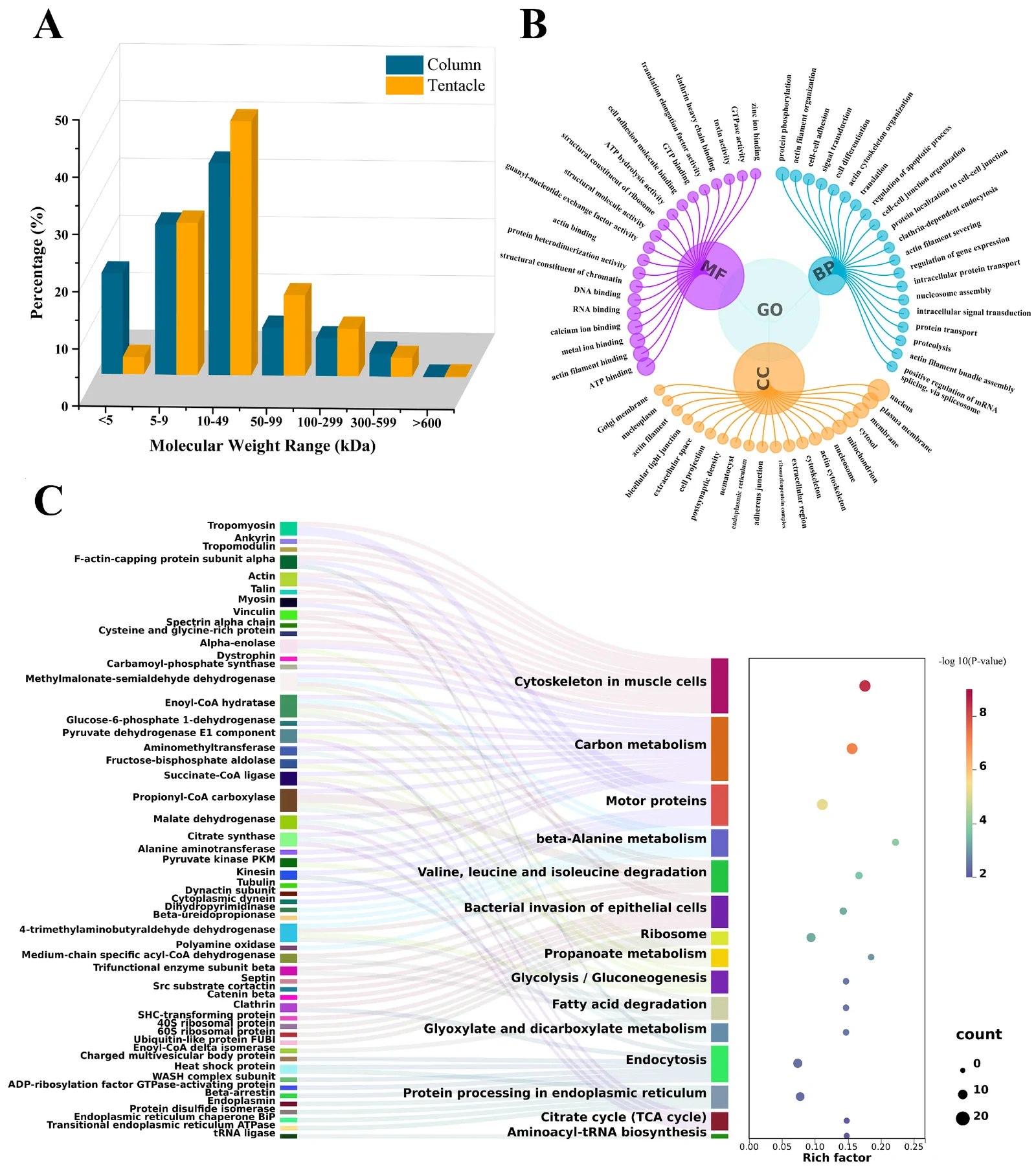
Partial analysis of identified proteins from proteomes of *H. magnifica*. (**A**) Distribution of the identified proteins with respect to their molecular weight and percentage of sequence coverage. (**B**) GO classification of the common proteins expressed between the column and the tentacle. Only those GO terms with at least 20 identified proteins are included. (**C**) Top fifteen KEGG pathways and their corresponding regulatory genes of the common proteins expressed between the column and the tentacle.

In order to further investigate the functional diversity of the proteins from *H. magnifica* and to establish a database to complement its transcriptome data, proteomic analysis was performed using LC-MS/MS technology. All putative proteins showed molecular weights ranging from 5 to more than 600 kDa using a database of protein sequences generated by Transdecoder from the transcriptome data of *H. magnifica* (Figure 2A). Of these proteins, 3846 proteins were found in the tentacle, 874 in the column, and 574 were common in both tissues (Supplementary Figure S2). The distribution of molecular weights and sequence coverage were similar between the two samples. Moreover, the sequences from the *H. magnifica* proteome were further subjected to BLAST (v2.17.0) searching and mapped for GO annotations, KEGG analysis and KOG database identification. The identified proteins from the column (Supplementary Figure S3), from the tentacle (Supplementary Figure S4), and those common to both (Figure 2B) were classified into the GO categories: biological processes (BP), cellular components (CC), and molecular functions (MF). As indicated in Figure 2B, the most represented GO terms in the BP category were protein phosphorylation, followed by actin filament organization and cell–cell adhesion. In the CC category, the most represented GO terms were nucleus, plasma membrane and membrane; whereas in the MF category, the most significant were ATP binding, actin filament binding and metal ion binding. In addition, the KEGG analyses of the column and the tentacle can be found in Supplementary Figures S5 and S6. Figure 2C reveals the top fifteen KEGG pathways and their corresponding regulatory genes of the common proteins between the two samples. The most prominent categories within the KEGG pathways were cytoskeleton-related pathways in muscle cells, carbon metabolism and motor proteins, providing valuable insights into the importance and interactions of these pathways in sea anemones. KOG database annotations further distributed 3578 unigenes from the tentacle and 735 unigenes from the column across 25 and 24 functional classes, respectively, with general function prediction and signal transduction mechanisms being the predominant categories (Supplementary Figures S7 and S8).

### 2.3. Annotation of Identified Proteins Detected in Both Transcriptome and Proteome

**Figure 3:**
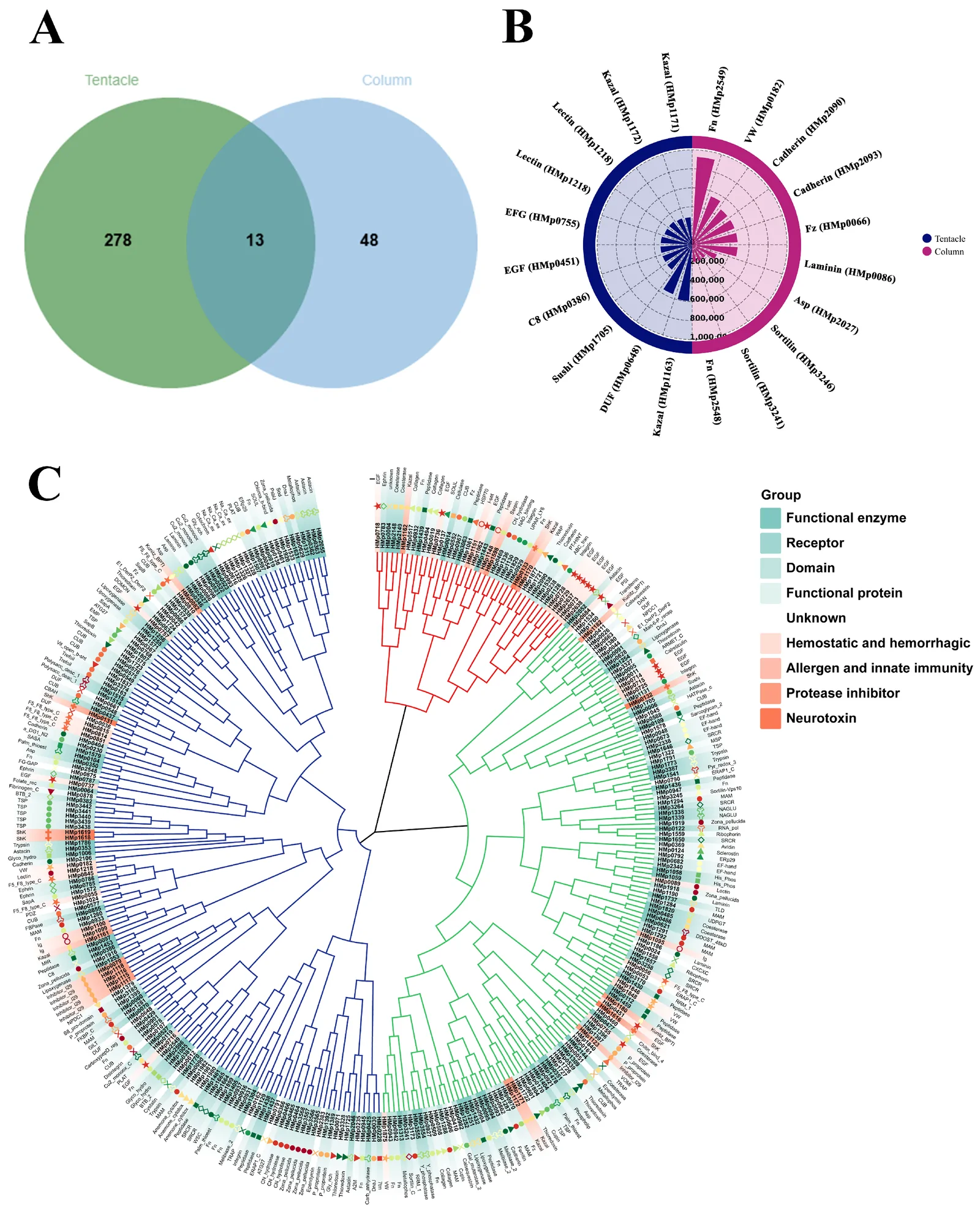
(**A**) Venn diagram of the 339 identified proteins detected in both the transcriptome and the proteome. (**B**) Ten proteins with the highest expression levels from the *H. magnifica* proteome. (**C**) Phylogenetic analysis of the 339 putative proteins using the Neighbor-Joining method (with 1000 bootstrap replicates). Symbols with different shapes denote distinct families.

A total of 339 proteins were identified, of which 278 were exclusively identified in the tentacle, 48 in the column, and 13 in both tissues (Figure 3A), following the manual remapping of 3846 proteins from the proteome database using transcriptome data. The low degree of overlap observed here is consistent with the transcriptome-based Venn diagram result (Supplementary Figure S1), supporting the functional specialization of proteins between the tentacles and the column. These 339 protein sequences were expressed in both the transcriptome and the proteome (Supplementary Table S4). Figure 3B reveals that the 339 proteins with the highest expression levels in the column were HMp2549 (Fn), HMp0182 (VW), HMp2090 (Cadherin), HMp2093 (Cadherin) and HMp0066 (Fz), whereas the proteins highly expressed in the tentacle were HMp1163 (Kazal), HMp0648 (DUF), HMp1705 (Sushi), HMp0386 (C8) and HMp0451 (EGF). Furthermore, the phylogenetic analysis of the 339 proteins is also shown in Figure 3C. Sequences with the same color indicate that the peptides are from the same biological function categories. The family of each protein is indicated by different symbols after the sequence name. All sequences are clustered into three major branches (clades I–III), with clade I containing 25 families, clade II 73, and clade III 98. Notably, the distribution of specific families across these clades exhibited distinct patterns: the EGF family predominantly clustered in clade I, EF-hand exclusively in clade II, and anemone_cytotox solely in clade III, whereas ShK and peptidase were widely distributed across all three clades. This suggests that while some functional families are evolutionarily constrained to specific lineages, others have undergone broader diversification or horizontal recruitment. In terms of functional category richness, clade I comprised eight functional categories, whereas clades II and III each contained nine, indicating a relatively balanced representation of biological functions across the major phylogenetic groups. However, it is important to note that although family assignments were primarily based on functional annotations, the phylogenetic grouping relied entirely on sequence similarity. Consequently, a given functional category may encompass families with divergent sequences, and conversely, sequences with high similarity do not necessarily share the same function. This decoupling between sequence identity and functional attributes underscores the complexity of venom protein evolution and highlights the necessity of integrating both phylogenetic and functional evidence for accurate protein characterization.

### 2.4. Exploration of the Functional Proteins from H. magnifica

#### 2.4.1. Functional Enzyme Category

This venom category accounted for the second-highest count of transcript clusters, including peptidase, trypsin, sulfatase, lipase, astacin, phospholipase, and so on (Figure 1). Peptidases constitute one of the most abundant groups of venom components, with a notable predominance of metalloproteases which are linked to hemorrhagic, necrotic, inflammatory, and tissue damage effects in the prey [12,13]. We identified ten families of metalloproteases in the transcriptome of *H. magnifica*. Among those, M1, M8 and M12A were present only in the tentacle and M2, M10, M13, M14, M23, M28, M12B were present in both the tentacle and the column (Supplementary Table S1). The M12A family is commonly documented in the venom of jellyfish, hydra and sea anemones [14,15,16], and is thought to play a significant role in breaking down the extracellular matrix [17].

**Figure 4:**
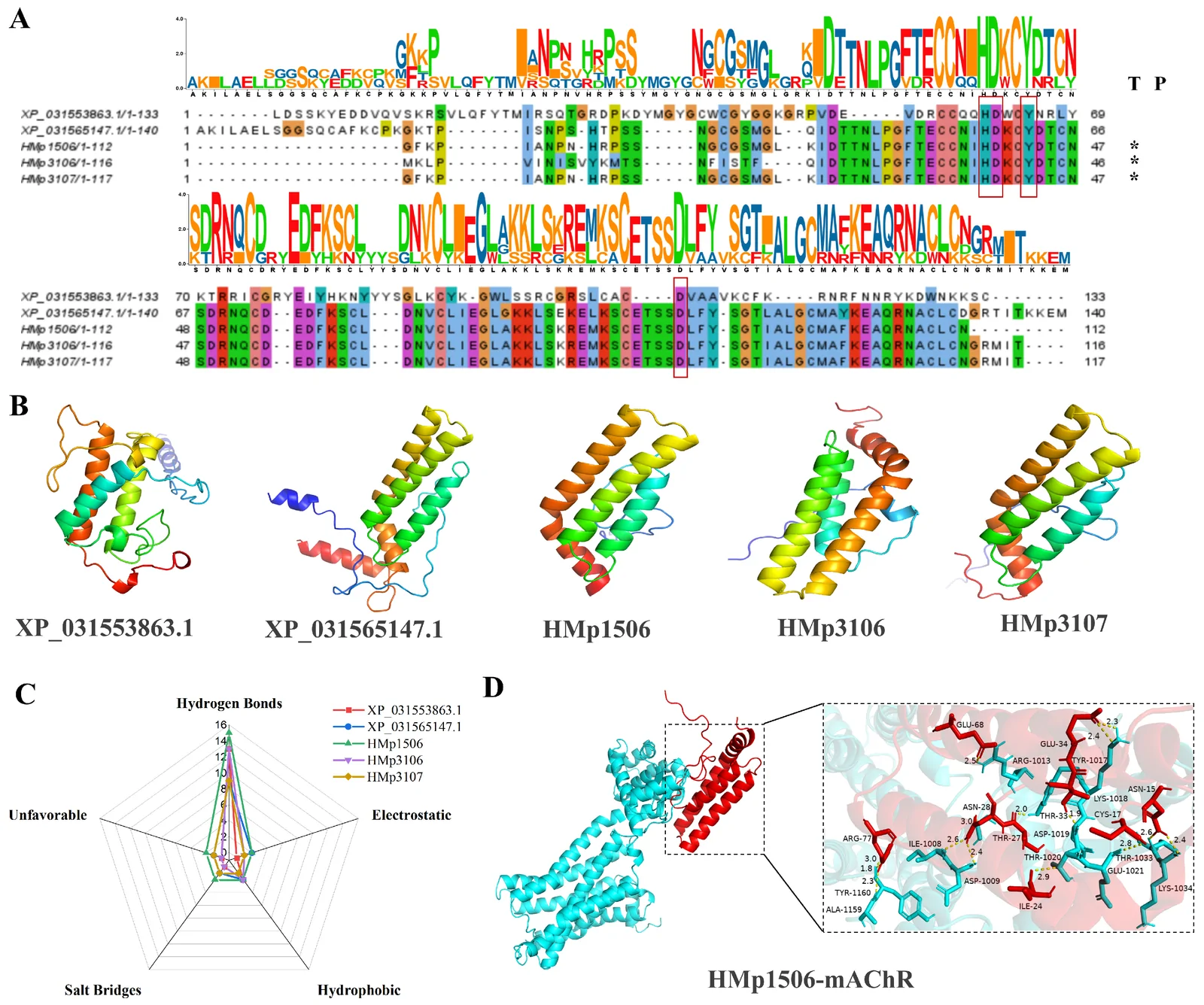
Putative PLA_2_ proteins from *H. magnifica*. (**A**) Multiple sequence alignment of known PLA_2_ proteins and representative PLA_2_ with mature regions using Jalview 2.11.5.2 and TBtools-II v2.390 under Clustal color scheme (T: transcriptome, P: proteomics; *: denotes sequences identified in the corresponding omics data). The representative PLA_2_ proteins shown are XP_031553863.1 (Uniport: A0A6P8HFV9) and XP_031565147.1 (Uniport: A0A6P8IED1) derived from *Actinia_tenebrosa*. The catalytic center characteristic of PLA_2_ is highlighted by red frame. (**B**) Structural models of mature region of these proteins predicted using AlphaFold3. (**C**) A radar chart illustrating the number of non-covalent interactions between PLA_2_ proteins and *Homo sapiens* mAChR (PDB: 5CXV). (**D**) Molecular docking analyses shows the binding interaction between HMp1506 and mAChR. The cyan color represents the receptor. The red color represents the HMp1506.

Moreover, another detected enzyme belongs to a phospholipase family known as phospholipase A_2_ (PLA_2_), which is more commonly found in almost all venomous animals [16], and exhibits a variety of pharmacological effects, including neurotoxicity, cardiotoxicity, myotoxicity, cytotoxicity and anticoagulant activity [18]. In cnidarians, these enzymes mainly contribute to digestive processes, defensive mechanisms, and hemolysis [19]. For instance, a PLA_2_ toxin with hemolytic activity was found in *Chrysaora* jellyfish [20], while the neurotoxic sPLA_2_ purified from sea anemone *Palythoa caribaeorum* was reported by a previous study [21]. In this study, three of the 23 PLA_2_ proteins were selected and their mature regions shared homologs with XP_031553863.1 and XP_031565147.1 from Australian red waratah sea anemone *Actinia tenebrousa* (Figure 4A). Notably, HMp1506, HMp3106 and HMp3107 exhibit high similarity to the reference sequence XP_031565147.1, with scores of 88.39%, 79.49% and 88.03% respectively. In addition, HMp1506 may possess catalytic activity, as it contains a catalytically active site, which agrees with the conserved catalytic residues of XP_031553863.1 confirmed by the previous literature [22]. As shown in Figure 4B, structure prediction indicated that all these proteins were predominantly α-helical. Moreover, selected PLA_2_ proteins exhibited the ability to inhibit the human M1 muscarinic acetylcholine receptor (mAChR) through different types of bonding interactions, including hydrogen bonds, salt bridge, electrostatic, hydrophobic, and other favorable bonds (Figure 4C). Among them, the protein with the highest number of hydrogen bonds is HMp1506 (15), followed by HMp3106 (13), whereas the two reference proteins, XP_031553863.1 and XP_031565147.1, possess 13 and 9 hydrogen bonds, respectively. Salt bridges with mAChR were formed exclusively by HMp1506 (2), HMp3107 (1), and XP_031565147.1 (1). As key non-covalent interactions, salt bridges are essential for maintaining structural stability and facilitating specific molecular recognition. Most proteins form 1–2 electrostatic and hydrophobic bonds with mAChR, whereas XP_031553863.1 lacks an electrostatic bond. Ultimately, HMp1506 was selected as the optimal protein among the candidates, and its binding interaction with mAChR is illustrated in Figure 4D. HMp1506 was predicted to form 21 favorable non-covalent bonds with mAChR, including 15 hydrogen bonds, e.g., ARG77-ALA1159; ARG77-TYR1160; ASN15-THR1033; ASN15-LYS1034; GLU68-ARG1013; THR33-ASP1019. Furthermore, the binding interfaces of HMp1506 and the two reference proteins on mAChR were all distinct from each other, indicating the absence of steric clashes (Supplementary Figure S9). This unique binding mode may confer improved receptor subtype selectivity or biased signaling.

#### 2.4.2. Functional Protein Category

**Figure 5:**
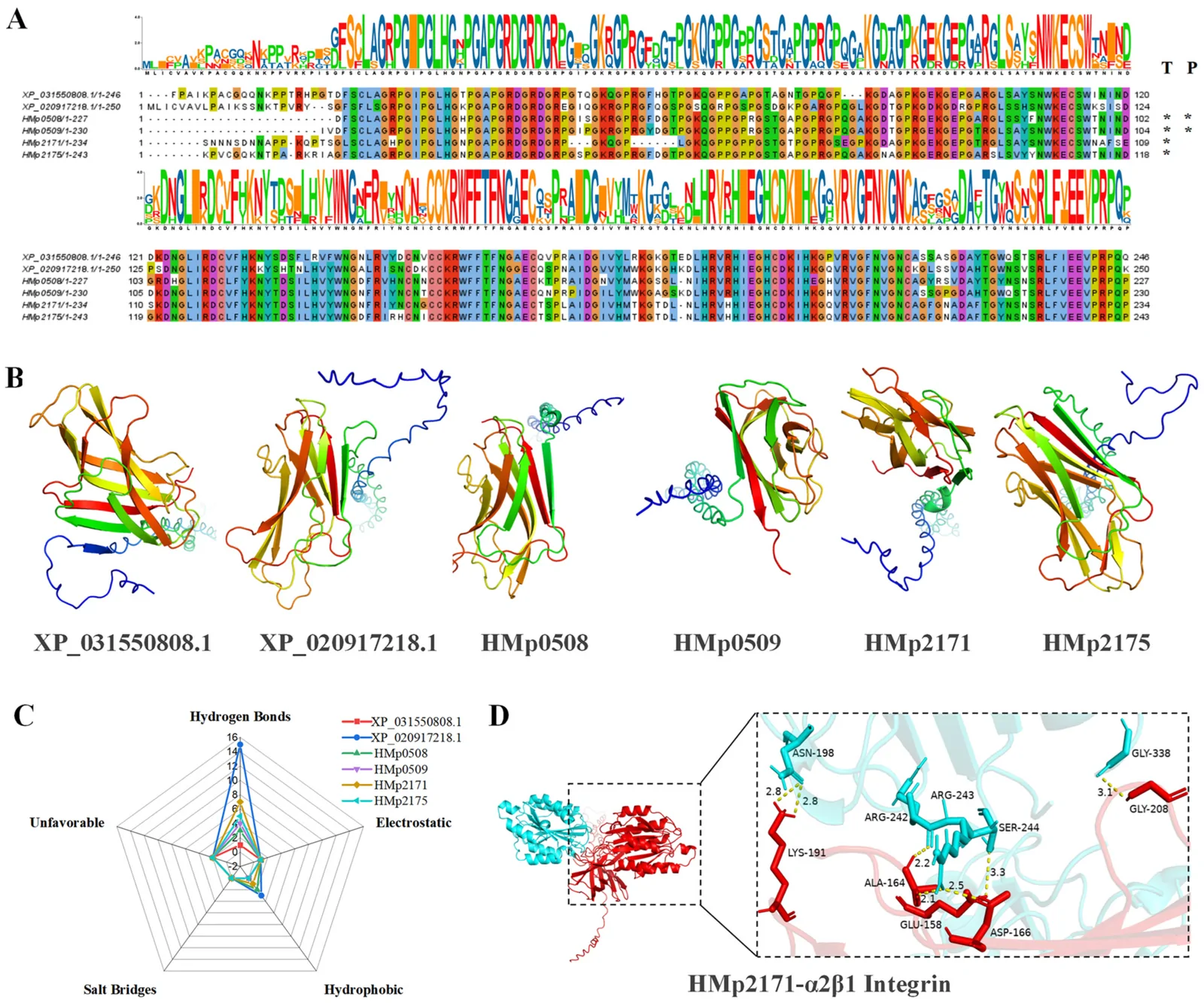
Putative collagens from *H. magnifica*. (**A**) Multiple sequence alignment of known collagens and representative collagen sequences with mature regions, generated using Jalview 2.11.5.2 and TBtools-II v2.390 under Clustal color scheme (T: transcriptome, P: proteomics; *: denotes sequences identified in corresponding omics data). The known collagens shown are XP_031550808.1 (Uniport: A0A6P8H5V9) from *Actinia tenebrosa*; XP_020917218.1 (Uniport: A0A913YA32) from *Exaiptasia diaphana*. (**B**) Structural models of mature region of these collagens predicted using AlphaFold3. (**C**) A radar chart illustrating the number of non-covalent interactions between collagens and the I-domain of human α2β1 integrin (PDB: 1AOX). (**D**) Molecular docking analysis shows the binding interaction between HMp2171 and α2β1 integrin. The cyan color represents the receptor. The red color represents the HMp2171.

This category encompasses a diverse array of high-molecular-weight functional proteins, including collagen, thioredoxin, fibrinogen_C, laminin, whey acidic protein (WAP), endomembrane protein 70 (EMP70), and so on. Collagen represents one of the most abundant and evolutionarily conserved protein families in cnidarian nematocysts, where it serves as a major structural component of extracellular matrixes and basement membranes [23]. In this study, 60 gene clusters annotated as collagen family were identified in the transcriptome (Figure 1A). Of these proteins, 24 were found only in tentacle, 31 only in the column, and 5 were present in both. Four representative collagens were closely matched to two known collagen-like proteins and were used to study their interactions with α2β1 integrin receptor (Figure 5). Integrin α2β1 is a widely distributed and functionally versatile collagen receptor that serves as a core mechanosensor and signaling hub in processes such as progenitor cell maintenance, tissue morphogenesis (platelet activation and thrombosis), and cancer metastasis [24]. For instance, researchers designed DGEA (a type I collagen-derived peptide that specifically binds α2β1) and attached it to liposomes, allowing precise transport of antitumor drugs to breast cancer cells overexpressing α2β1 [25]. The sequences alignment and structure prediction are performed in Figure 5A and 5B. Four selected collagens shared homology with collagen identified in *Actinia tenebrosa* (XP_031550808.1) and *Exaiptasia diaphana* (XP_020917218.1). As shown in Figure 5C, six selected collagens exhibited different degrees of binding to the I-domain of the α2β1 integrin, since I-domain of α2 subunit had been regarded as the major collagen-binding site of α2β1 integrin [26]. However, one study exceptionally found that a collagen-like snake venom protein (Trimucytin) isolated from *Trimeresurus mucrosquamatus* may interact with other reactive site(s) expressed on α2β1 integrin [27]. Its mechanism of action has also updated our traditional understanding of the functional domains of this receptor. According to the radar chart, XP_020917218.1 exhibited the greatest number of hydrogen bonds (15), with HMp2171 ranking second (7). However, all collagens formed only one electrostatic interaction and lacked salt bridges. The molecular docking analysis of HMp2171 with α2β1 integrin is shown in Figure 5D, with hydrogen bonds including LYS191-ASN198, ALA164-ARG242, ALA164-ARG243, ASP166-ARG243, GLU158-SER244, and GLY208-GLY338. As illustrated in Supplementary Figure S10, HMp2171 bound α2β1 integrin at a site adjacent to that of reference collagen XP_020917218.1, but its binding site was spatially distant from that of XP_031550808.1, which formed only one hydrogen bond with the receptor. On the other side, this protein shared similar sequence identities with the two reference proteins, which are 69.07% and 66.25%, respectively. These findings suggested that while sequence similarity did not necessarily reflect binding site location or interaction strength, the number of hydrogen bonds might provide a valuable structural measure of binding affinity.

Of particular note, an Hsp70 protein (HMp1061) discovered in the tentacle transcriptome was assigned to the heat shock protein (HSP) family, which facilitates protein folding, prevents aggregation, and helps reestablish protein homeostasis within the cell [28]. This protein shares over 96% identity with Hsp70 sequences from other sea anemones (*Actinia tenebrosa* and *Nematostella vectensis*). Additionally, this protein was also detected in the proteome, and its expression was confirmed at the protein level. It has been reported that Hsp70 exhibits a range of potential pharmacological activities, including functioning as a carrier for tumor-derived immunogenic peptides to induce T cell-mediated anticancer immunity, exerting neuroprotective effects in mammals, and preventing the oligomerization of amyloidogenic proteins [29].

#### 2.4.3. Hemostatic and Hemorrhagic Category

**Figure 6:**
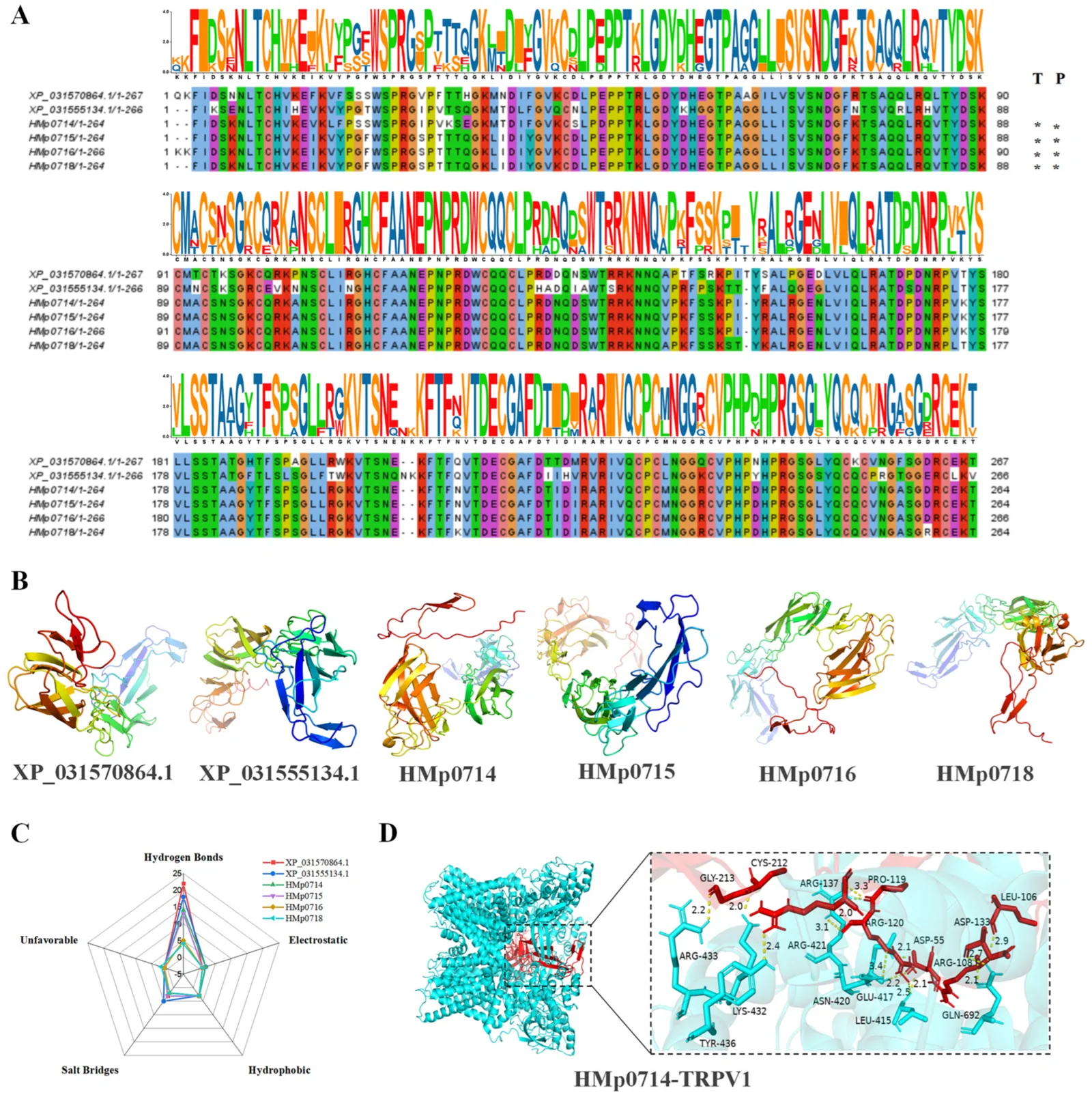
Putative EGF-like proteins from *H. magnifica*. (**A**) Multiple sequence alignment of the mature peptide core regions of known EGF toxins and representative EGF proteins using Jalview 2.11.5.2 and TBtools-II v2.390 under the Clustal color scheme (T: transcriptome, P: proteomics; *: denotes sequences identified in the corresponding omics data). The known EGF-like toxins shown are XP_031570864.1 (Uniport: A0A6P8IUZ9) and XP_031555134.1 (Uniport: A0A6P8HFE1) from *Actinia tenebrosa*. (**B**) Structural models of mature peptide core regions of these EGF-like proteins predicted using AlphaFold3. (**C**) A radar chart illustrating the number of non-covalent interactions between EGF-like proteins and Homo sapiens TRPV1 channel (PDB: 8GFA). (**D**) Molecular docking analysis shows the binding interaction between HMp0714 and TRPV1. The cyan color represents the receptor. The red color represents the HMp0714.

As prevalent venom components with potential pharmacological activity, the hemostatic and hemorrhagic proteins found in this study were assigned largely to five toxin protein families: epidermal growth factor (EGF), Coagulation factors V and VIII type C domain (F5_F8_type_C), von Willebrand factor (VW), lectin and FXa_inhibition. These families have been well documented among cnidarians and are known to disrupt prey hemostasis through distinct mechanisms, including proteolytic degradation of coagulation factors, disruption of platelet aggregation, and damage to vascular endothelium [30]. As the dominant family of this category, 32 sequences annotated as EGF-like domain-containing proteins (EGF-like proteins) were identified in the column and 87 sequences were identified in the tentacle (Figure 1). Among them, four representative EGF-like proteins shared homology with two EGF domain-containing proteins identified in *Actinia tenebrosa*, as shown in Figure 6A. These EGF proteins were all selected from the aforementioned set of 339 proteins (Part 2.3), which were identified by both transcriptomic and proteomic analyses. Homology modeling revealed that these proteins adopt a β-sheet-dominated secondary structure, with the sheets connected by loop regions (Figure 6B). All EGF-like proteins could interact with TRPV1 (transient receptor potential vanilloid 1) receptor, as depicted in Figure 6C,D. TRPV1, a member of the TRP ion channel family, responds to diverse noxious stimuli such as temperature changes, pH variations, and irritant compounds [19]. This channel is a key mediator of nociception and inflammation, and is overexpressed in chronic inflammation and neuropathic pain [31]. Some EGF-like peptides derived from cnidarians, such as *Stichodactyla giganteus* and *Exaiptasia diaphana*, have been identified as inhibitors or modulators of the TRPV1 channel [23,32]. A case in point is HCRG21, a jellyfish venom-derived peptide that strongly modulates TRPV1, and markedly dampened the inflammatory response by blocking capsaicin-triggered activation [33]. In this study, six selected EGF-like proteins with high molecular weights could bind to the TRPV1 channel. Notably, the four proteins—XP_031570864.1, XP_031555134.1, HMp0714 and HMp0715—formed 22, 18, 14, and 13 hydrogen bonds and 3, 5, 2, and 3 salt bridges with the receptor, respectively. In addition to the reference proteins, the interaction of HMp714—selected as the best candidate—with TRPV1 is shown in Figure 6D. The 21 favorable non-covalent bonds between HMp0714 and TRPV1 include 14 hydrogen bonds, e.g., GLY213-ARG433; CYS212-LYS432; ARG137-TYR436; ARG120-ARG421; LEU106-GLN692; ASP133-GLN692. The binding site is located in the central ion channel of the receptor, coinciding with the binding sites of the two reference proteins (Supplementary Figure S11). These TRPV1-modulating EGF-like proteins not only serve as leads for non-opioid pain relief and anti-inflammation but also provide structural scaffolds for drug design and pharmacological probes to study TRPV1-related pathologies.

We also found numerous venom prothrombin-related factors in two groups: F5_F8_type_C and von Willebrand factor (VW), as shown in Figure 1. These toxins are well known from snake venoms to cause hemostatic impairment by activating prothrombin or by modulating platelet aggregation [34]. As depicted in Supplementary Figure S12, we selected eight representative proteins from the F5_F8_type_C family and these proteins exhibit a 25–36% identity with A7RK24 which is the known F5/8 type C domain-containing protein from Starlet sea anemone *Nematostella vectensis* [3]. These sequences represent a potential source for the discovery of novel drugs targeting thrombosis or coagulation. A smaller subset of transcripts from this category of *H. magnifica* was also identified, including the FXa inhibition, hemopexin and fibroblast growth factor (FGF) family. For example, two representative transcripts from the FXa inhibition family showed similarity to P00742 from *Homo sapiens* and XP_031553062.1 from *Actinia tenebrosa* (Supplementary Figure S13).

#### 2.4.4. Allergen and Innate Immunity Category

**Figure 7:**
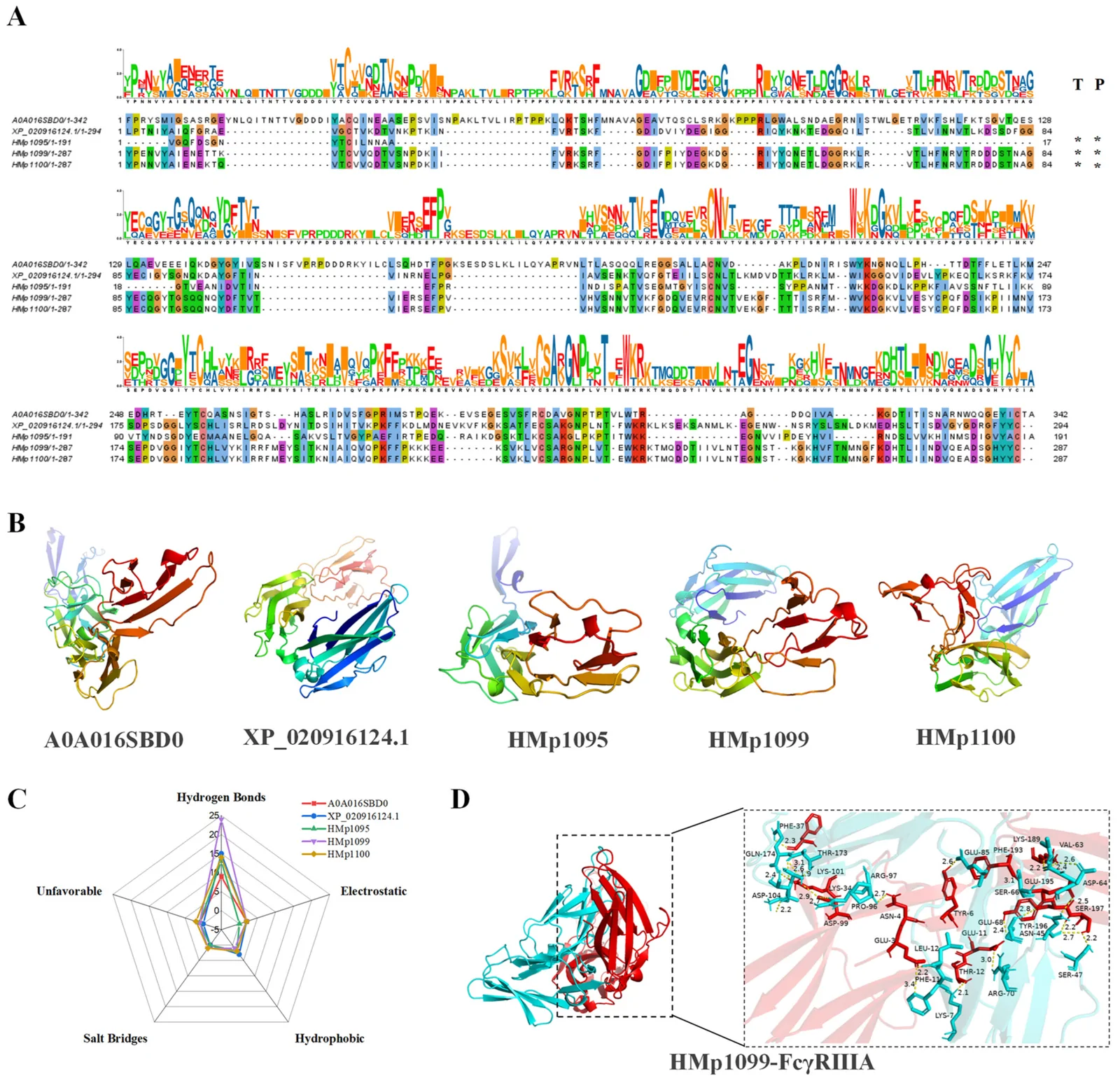
Putative Ig-like proteins from *H. magnifica*. (**A**) Multiple sequence alignment of known Ig protein and representative Ig proteins with mature peptide core regions using Jalview 2.11.5.2 and TBtools-II v2.390 under Clustal color scheme (T: transcriptome, P: proteomics; *: denotes sequences identified in the corresponding omics data). The known EGF toxins shown are A0A016SBD0 from *Ancylostoma ceylanicum* and XP_020916124.1 (Uniport: A0A913Y8Y1) from *Exaiptasia diaphana*. (**B**) Structural models of mature peptide core regions of these Ig proteins predicted using AlphaFold3. (**C**) A radar chart illustrating the number of non-covalent interactions between Ig proteins and Homo sapiens FcγRIIIA receptor (PDB: 3SGJ). (**D**) Molecular docking analyses shows the binding interaction between HMp1099 and FcγRIIIA receptor. The cyan color represents the receptor. The red color represents the HMp1099.

Transcripts annotated as allergens and innate immunity genes are widespread in the venom arsenals of reptiles, arthropods and cnidarians [1,35]. In this study, 102 protein sequences were identified in the column transcriptome and were mainly classified into four major families: Ig (69), I-set (19), CAP (13), and Bcl-2 (1) (Figure 1). In addition, 76 protein sequences were identified in the tentacle transcriptome, including Ig (53), I-set (16), CAP (5) and Grp7_allergen (2). Immunoglobulin family proteins, including members with Ig and I-set domains, represent the most abundant immune-related components in both column and tentacle transcriptomes. The FcγRIIIA receptor is a transmembrane glycoprotein expressed on natural killer cells, monocytes, and macrophages, serving as a receptor for the constant Fc fragment of immunoglobulin [36]. FcγRIIIa endows NK cells with a unique capacity to recognize and clear tumor cells opsonized by IgG, reflecting an evolutionary adaptation dedicated to antitumor immunity [37]. In this study, three transcripts annotated as Ig proteins were selected and showed approximately 34% identity to known mature Ig proteins, with members of this family present in the nematode *Ancylostoma ceylanicum* and sea anemone *Exaiptasia diaphana* (Figure 7A). Three Ig proteins were selected from the 339 proteins database identified by both transcriptome and proteome analyses. AlphaFold3 was used to predict the structures of identified proteins and known Ig proteins, as illustrated in Figure 7B. All proteins possess the typical antiparallel β-sheet structure characteristic of immunoglobulins. As depicted in Figure 7C, a large-scale molecular docking analysis of five Ig-like proteins with the FcγRIIIa receptor was conducted to investigate their potential interactions and biological relevance. It is worth noting that all proteins formed a large number of hydrogen bonds with the FcγRIIIa receptor, with HMp1099 showing the highest count (24), followed by XP_020916124.1 (15), HMp1100 (14), and HMp1095 (13), and finally A0A016SBD0 (9). The specific amino acids involved in the hydrogen bonds (e.g., GLU11-ARG70; THR12-LYS7; LYS189-VAL63; LYS189-ASP64; LYS34-GLN174; LYS34-THR173) formed between HMp1099 and FcγRIIIa receptor, are shown in Figure 7D. According to Supplementary Figure S14, HMp1099 was bonded to the same site on the FcγRIIIa receptor as reference protein A0A016SBD0, whereas its binding site is different from that of the other reference protein. These results raise the possibility that Ig proteins in sea anemones modulate inflammatory responses or disrupt host immune surveillance through interactions with the FcγRIIIA receptor.

Venom CAP proteins have broad roles, ranging from ion channel modulation and inflammation to proteolysis and immune regulation [38]. Snake CRISPs, for instance, inhibit channels and angiogenesis, boost vascular leakage, and recruit leukocytes, fueling local damage and inflammation [39]. CAP-domain proteins are also found in sea anemone venoms (e.g., *Bunodosoma caissarum*, *Actinia fragacea*), often mixed with neurotoxins and cytolysins, suggesting they play a role in prey capture or defense [22,40]. In *H. magnifica*, 19 CAP-domain proteins were revealed, including 14 sequences expressed in the column and five sequences expressed in the tentacle. The Bcl-2 family, although represented by only two sequences in the column transcriptome of *H. magnifica*., warrants attention due to its well-established role in apoptosis regulation. Snake venom toxins frequently trigger apoptosis in cancer cells by altering the balance of Bcl-2 family members—e.g., by downregulating anti-apoptotic Bcl-2 and/or upregulating pro-apoptotic Bax—as reported in breast [41], colon [42], and neuroblastoma cell lines [43]. According to these findings, the presence of Bcl-2 proteins in sea anemone venom suggests they might induce cell death in prey or interfere with predator immunity.

#### 2.4.5. Protease Inhibitor Category

**Figure 8:**
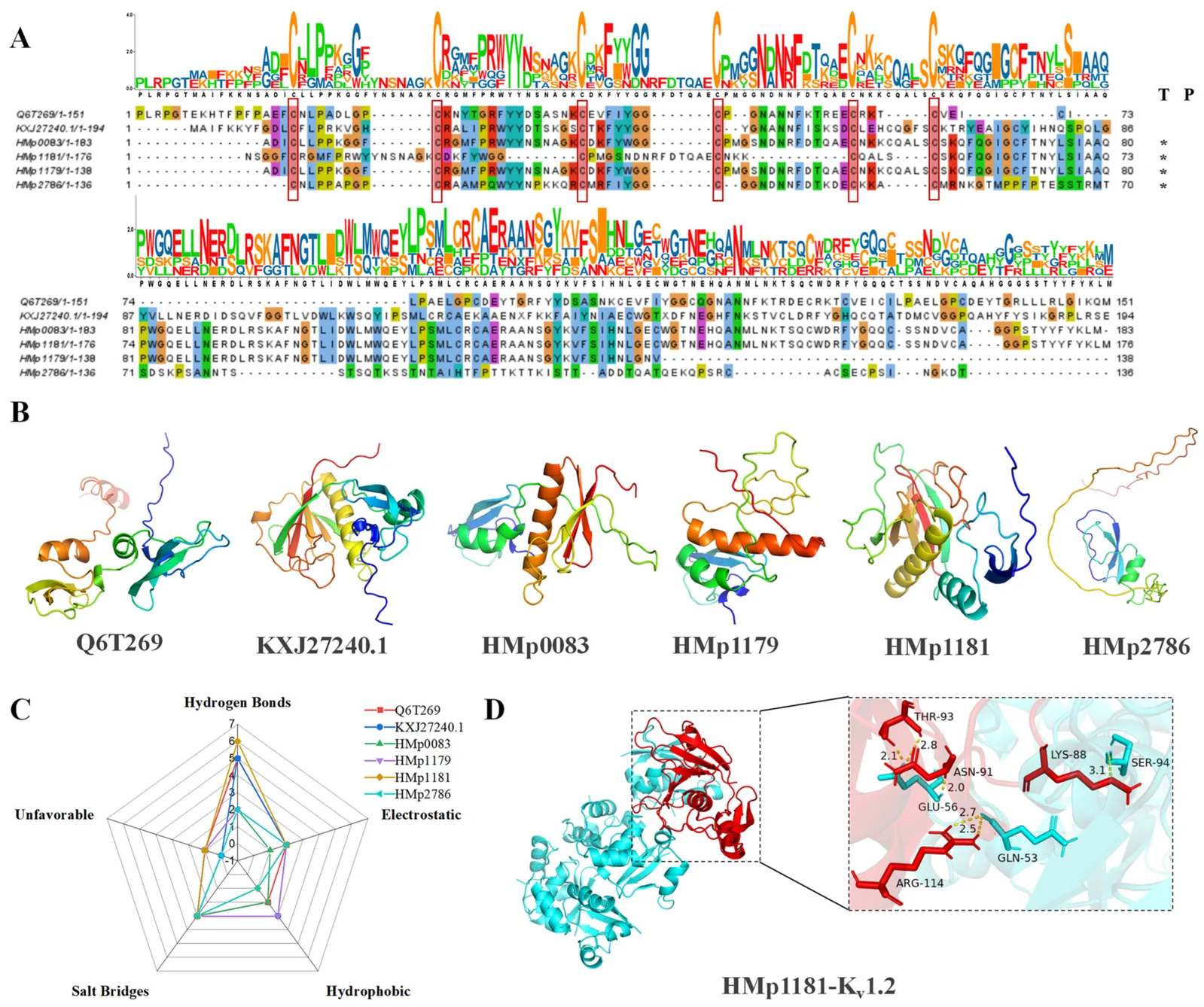
Putative Kunitz-type proteins from *H. magnifica*. (**A**) Multiple sequence alignment of known Kunitz-type proteins and representative Kunitz-type proteins with mature regions using Jalview 2.11.5.2 and TBtools-II v2.390 under Clustal color scheme (T: transcriptome, P: proteomics; *: denotes sequences identified in the corresponding omics data). The representative Kunitz-type proteins shown are Q6T269 from *Bitis gabonica* and KXJ27240.1 from *Exaiptasia diaphana*. The cysteine signature of all proteins is highlighted by red frame. (**B**) Structural models of mature region of these proteins predicted using AlphaFold3. (**C**) A radar chart illustrating the number of non-covalent interactions between Kunitz-type proteins and the K_v_1.2 channel (PDB:1DSX). (**D**) Molecular docking analyses shows the binding interaction between the Kunitz-type protein HMp1181 and the K_v_1.2 channel. The cyan color represents the receptor. The red color represents the HMp1181.

In this work, the protease inhibitor category in *H. magnifica* venom encompasses several additional structurally and functionally distinct families, including Kunitz-type proteins, Kazal-type inhibitors, serpins, and thyroglobulin type-1 domain proteins. Kunitz-type proteins have been recruited as venom components across a multitude of taxa—including snakes, scorpions, spiders, cone snails, ticks, and sea anemones—where they play pivotal roles in prey immobilization, predator deterrence and the disruption of hemostatic systems [44]. Kunitz-type proteins exert their physiological and toxic effects primarily through two distinct and well-characterized pathways: canonical inhibition of serine proteases [45] and allosteric modulation of voltage-gated potassium ion channels (K_v_) [46]. Although Kunitz-type inhibitors are generally small with a length of approximately 60 amino acids, 15 transcripts encoding high-molecular-weight members of this family (80–200 amino acids) were found in *H. magnifica* in this study. Among the Kunitz-type proteins identified in *H. magnifica*, four transcripts with six conserved cysteine residues showed similarity to Q6T269 from the snake *Bitis gabonica* and KXJ27240.1 from the sea anemone *Exaiptasia diaphana* (Figure 8A). Homology modeling revealed that all proteins contain a Kunitz domain, featuring both antiparallel β-sheets and α-helical regions (Figure 8B). The radar chart showed that HMp1181, Q6T269, and KXJ27240.1 formed 6, 5, and 5 hydrogen bonds with K_v_1.2 channel, respectively. Moreover, all three complexes contained three salt bridges and two electrostatic bonds (Figure 8C). Ultimately, HMp1181 was selected as the optimal protein among the candidates, and its binding interaction with Kv1.2 channel is illustrated in Figure 8D. Docking analysis revealed that HMp1181 exhibited a favorable binding profile to the Kv1.2 channel, forming six hydrogen bonds, including LYS88-SER94; ARG114-GLN53; THR93-GLU56; ASN91-GLU56. In addition, protein HMp1181 was identified as homologous to Q6T269 (42.86%) and KXJ27240.1 (47.98%). As depicted in Supplementary Figure S15, HMp1181 and the two reference proteins shared the same binding site on the K_v_1.2 channel, suggesting a possible inhibitory influence on the channel.

Beyond Kunitz-type proteins, four transcripts corresponding to the serpin family of serpin protease inhibitors were identified in this work in both tentacle and column transcriptomes of *H. magnifica*. Similarly, this type of protein has been found in the crude venom extracts of both *Anthopleura dowii* and *Lebrunia neglecta*, which may serve as models for designing serpin-based therapeutics for inflammatory diseases such as rheumatoid arthritis, owing to their high sequence similarity to human serpin B6 (which inhibits cathepsin G and trypsin) [29,47]. In addition, five thyroglobulin type-1 domain-containing proteins were also found in both the tentacle and column transcriptomes of *H. magnifica*. Equistatin, discovered from *Actinia equina*, is a cysteine endopeptidase inhibitor sequence with 199 amino acid residues distributed in three thyroglobulin type 1 domains [3], which can inhibit cathepsin D and cathepsin B, making it a tool for studying lysosomal proteases and a potential candidate for treating conditions involving aberrant cathepsin activity (e.g., tumor metastasis and osteoarthritis) [48].

#### 2.4.6. Neurotoxin Category

**Figure 9:**
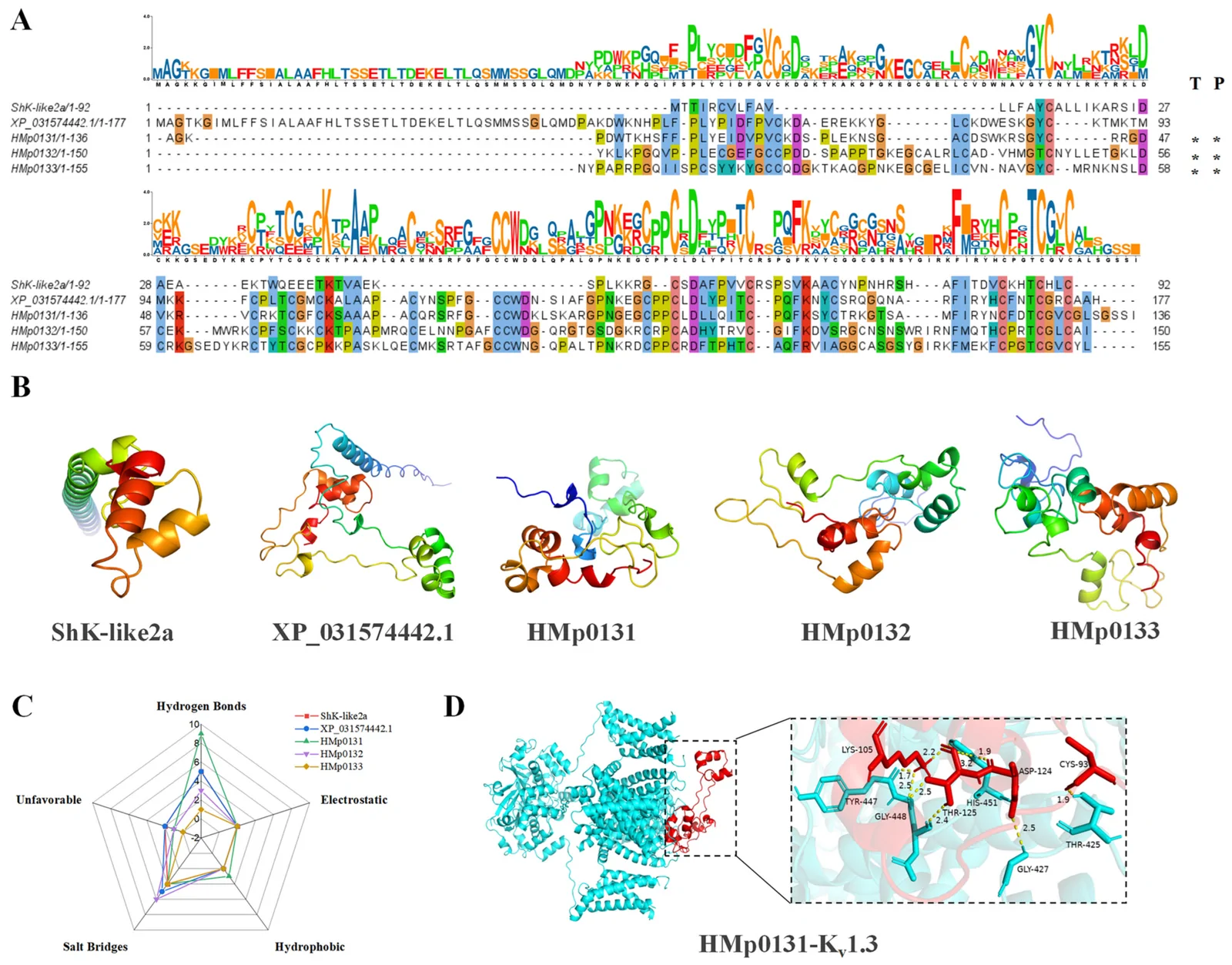
Putative ShK proteins from *H. magnifica*. (**A**) Multiple sequence alignment of known ShK-like proteins and representative ShK proteins with mature regions using Jalview 2.11.5.2 and TBtools-II v2.390 under Clustal color scheme (T: transcriptome, P: proteomics; *: denotes sequences identified in the corresponding omics data). The representative ShK-like proteins shown are ShK-like2a (Uniport: A7SRR3) from *Nematostella vectensis* and XP_031574442.1 (Uniport: A0A6P8J454) from *Actinia_tenebrosa*. (**B**) Structural models of mature region of these proteins predicted using AlphaFold3. (**C**) A radar chart illustrating the number of non-covalent interactions between ShK proteins and the K_v_1.3 channel (PDB: 7ssx). (**D**) Molecular docking analyses shows the binding interaction between the ShK proteins HMp0131 and the K_v_1.3 channel. The cyan color represents the K_v_1.3 channel. The red color represents the HMp0131.

Neurotoxins are a series of toxins that directly target the ion channels of cell membranes, leading to interference with the nervous and muscular systems of prey and predators. This category is consistently dominated by ShK-like toxins containing peptides and proteins. Typically, ShK-like toxins consist of a signal peptide and a region of the mature protein composed of less than 60 amino acid residues, as reported in previous studies [3,10]. On this basis, the present work focused on the discovery of ShK-like toxins with high-molecular-weight. In the transcriptome of *H. magnifica*, 19 column gene clusters and 22 tentacle gene clusters were respectively assigned as ShK-like protein family members belonging to the neurotoxins category (Figure 1A). Three of these ShK-like proteins were selected for comparative analysis, which were expressed both in transcriptome and proteome (Figure 9A). Normally, a typical secondary structural core of classical ShK domain peptides consists of 2–3 α-helices. In this study, AlphaFold3 was used to predict the structural models of these ShK proteins, and the results were consistent with the above structural paradigm (Figure 9B). Generally, ShK-like neurotoxins are widely considered to be inhibitors of the K_v_ channel. Homologous modeling and molecular docking analysis demonstrated that all models successfully docked with the K_v_1.3 channel (Figure 9C). The five neurotoxins—HMp0131, ShK-like2a, XP_031574442.1, HMp0132 and HMp0133—formed 9, 5, 5, 3 and 1 hydrogen bonds and 4, 4, 5, 6 and 4 salt bridges with the receptor, respectively. In addition, electrostatic and hydrophobic bonds were each counted at 2–3 per complex. Among the successfully docked proteins, HMp0131 exhibited the best docking performance, forming nine hydrogen bonds, e.g., CYS93-THR425; ASP124-GLY427; THR125-HIS451; THR125-GLY448; LYS105-TYR447; LYS105-HIS451 (Figure 9D). This sequence showed 60% identity with XP_031574442.1 from sea anemone *Actinia tenebrosa*. In addition, HMp0131 interacted with the K_v_1.3 channel at a site directly above the pore, whereas the two reference proteins bonded to the opposite side of the pore (Supplementary Figure S16). Nevertheless, all three proteins were able to block ion permeation through the channel, thereby exerting an inhibitory effect.

#### 2.4.7. Other Category

Other groups of proteins identified in *H. magnifica* fall into the domain, receptor and unknown category. Among them, domain category contained the highest number of transcripts which comprise CUB, Fn, Tsp, Fz, cadherin and other domain families. Beyond the domain category, 159 transcripts in tentacle and 175 transcripts in the column that were assigned to the unknown venom category. These proteins resemble those in other categories but have unknown functions or modes of action. For example, four transcripts corresponding to the family of blomia tropicalis allergen 5 (Blo-t-5) were detected in the tentacle transcriptome but were not identified in the proteome of *H. magnifica*. To our knowledge, this is the first report of Blo-t-5 in the sea anemone. Blo-t-5 possesses a unique three-helix bundle structure that specifically binds to IgE antibodies, thereby triggering allergic reactions. As a biomarker, Blo-t-5 holds significant value in clinical diagnostics and has also demonstrated potential for application in immunotherapy in asthma animal models [49,50].

## 3. Discussion

Using transcriptome and proteomics, this study systematically classified the proteins of the sea anemone *H. magnifica* into functional families for the first time and explored their potential physiological activities through sequence alignment, homology modeling, and molecular docking analysis. As shown in Figure 1, the transcriptome identified 3573 putative proteins sequences, comprising 1925 and 1835 from the tentacle and the column. These sequences were categorized into nine functional categories. Remarkably, we also found significant differential protein expression between the tentacle and the column, and this divergence is highly consistent with their respective physiological and ecological functions, which is in good agreement with previous reports on tissue-specific venom component distribution in sea anemones [10,22]. Some families were shared between the two tissues, yet they may differ in both abundance and functional roles. For example, our study showed that the tentacle of *H. magnifica*—a sensitive and easily damaged locomotory organ—exhibited marked EGF enrichment, which is associated with cell proliferation and tissue regeneration, suggesting that its high EGF levels may contribute to its remarkable regenerative capacity. In contrast, EGF enriched in the column may primarily contribute to structural support and extracellular matrix maintenance, which remains to be directly verified by further functional experiments.

In addition, proteomics analysis of identified proteins of *H. magnifica* was performed, and 339 proteins were found in both the transcriptome and proteome after manual remapping the proteins from proteome database using transcriptome data. It is worth noting that 21 proteins belonging to six typical protein families were identified; their structures were predicted, and their interactions with proposed targets were also analyzed. Among them, some proteins were part of 339 proteins detected in both datasets, which strongly indicates that the corresponding gene is genuinely expressed, translated into protein, and present at reasonably high levels, thereby increasing their credibility as functional venom components. Within the nine functional categories of *H. magnifica*, each was characterized by representative families with high transcript abundance, such as peptidase, collagen, EGF, F5_F8_type_C, Ig and CUB domain. These highly abundant families were likely to underpin the principal physiological functions and can serve as primary candidates for further bioprospecting efforts.

One of the most numerous groups within the functional protein category was collagen (total 60), which were comparatively highly abundant in *H. magnifica*. A transcriptomic study of *Exaiptasia diaphana* across developmental stages identified collagen, along with metalloproteases, chymotrypsinogen-like proteins, and GPCRs, as major predicted toxin superfamily components [23]. Collagen expression peaked at the middle stage and remained high in mature animals, supporting its correlation with adhesion and structural demand during growth. In general, the repetition of the Gly-Pro-X tripeptide generates a (Gly-Pro-X)_n_ pattern (where n represents the number of repeats), which is considered a hallmark of nematocyst structural collagens [51] and is exceptionally stable and resistant to degradation by common proteases [52]. The collagens derived from *H. magnifica* also contained this conserved domain architecture, as shown in Figure 5. Beyond their roles in adhesion and structural demand, few collagen-like proteins have also been explored as molecular tools to study platelet receptors. One example is trimucytin, which has been used as pharmacological probes to dissect the role of integrin α_2_β_1_ in thrombosis and hemostasis [27]. These findings underscore the utility of venom-derived collagen in both basic research and therapeutic applications.

As for the Ig family, the interaction between FcγRIIIa receptor and Ig proteins derived from sea anemone was investigated by molecular docking analysis for the first time. Although direct evidence for sea anemone Ig-like proteins interacting with Fcγ receptors remains limited, structural and functional parallels (C-type lectin) can be drawn from snake venom systems. For instance, convulxin derived from *Crotalus durissus terrificus* activates platelets by binding to the GPVI/FcR γ-chain complex, triggering tyrosine phosphorylation and downstream signaling [53,54]. Similarly, the snake venom lectin mucetin activates platelets via GPIb and triggers the translocation of the Fc receptor γ chain to the cytoskeleton, further supporting the concept that venom proteins exploit immune receptor-associated signaling cascades [55]. Both cases reveal how venom components can commandeer immune receptor-associated signaling to control platelet function. These studies provide a reference for the role of Ig proteins in sea anemones in regulating inflammatory responses through interactions with components of the FcγRIIIa receptor signaling pathway. However, this hypothesis requires experimental validation.

This study also uncovered several low-abundance yet potentially important proteins, including insulin-like, heat shock proteins, translin, Blo-t-5, and Ricin_B_lectin, which may expand our understanding of venom complexity. Few insulin-like proteins were identified in sea anemone [1], although some insulin-like peptides were found in *Exaiptasia diaphana* and *Nematostella vectensis,* which were either similar to human insulins or belong to the cono-insulins family from cone snail toxins [23,56]. The insulin-like venom proteins HMp2735 and HMp2736, that we obtained from the column transcriptome of *H. magnifica*, were reported here for the first time. These two insulin proteins showed modest sequence similarity to human insulin (UniProt ID: P01308) and sea anemone-derived insulin (UniProt ID: A0A6P8HRZ6 and A0A6P8J6M1). We also identified one protein (HMp3372) from the column transcriptome that belongs to translin family, which has been found in *Nematostella vectensis*. The translin homolog identified in the genome of *Nematostella vectensis* (UniProt ID: A7SQQ6) was derived from the NEMVEDRAFT_v1g215929 genomic locus, as annotated within the framework of the starlet sea anemone genome project [57]. Gupta et al. systematically summarized the multiple functions of translin in nucleic acid metabolism and its association with cancer [58]. In certain Dicer-deficient cancer cells, the translin–TRAX complex degrades tumor-suppressive microRNA precursors, thereby promoting tumorigenesis and emerging as a potential therapeutic target [59]. In addition, one of eight avidin proteins (HMp0369) identified in the *H. magnifica* transcriptome was detected by proteomics, confirming its expression at the protein level and suggesting its potential role as a functional venom component. Avidin plays key roles in oviparous vertebrates by binding biotin with high affinity to mediate antimicrobial defense and nutritional regulation [60]. A domain of the sea urchin, Fibropellin protein shows sequence similarity to avidin/streptavidin, but no clear evidence confirms the presence of avidin proteins in sea anemones [61]. This study provides a clue to the existence of avidin-like proteins in cnidarians, highlighting an underexplored family with potential fundamental biological functions. Notably, another protein matched to a neurotoxin called ricin_B_lectin was first identified in the transcriptome of *H. magnifica*. It is originally characterized in the plant toxin ricin from *Ricinus communis*, but subsequently in bacterial insecticidal toxins [62]. Ricin_B_lectin can bind specifically to galactose and other cell-surface sugars, acting as a molecular handle for toxin attachment to the target membrane.

In summary, the integrated transcriptomic and proteomic profiling of *H. magnifica* presented here has substantially expanded the known protein repertoire of this sea anemone, revealing not only major toxin families—such as metalloprotease, Kunitz-type inhibitor, ShK-like neurotoxin, and collagen—but also previously unreported or low-abundance proteins, including insulin-like peptides, translin, avidin-like proteins, and Blo-t-5. Homology modeling and molecular docking analyses have provided initial structural and functional clues for several of these proteins, particularly regarding their potential interactions with integrins, ion channels, and immune receptors. However, these in silico predictions await experimental validation through functional assays, such as electrophysiological recordings, platelet aggregation tests, and in vivo toxicity studies, to confirm their actual pharmacological activities. Moreover, the potential synergistic effects among these venom components and their evolutionary dynamics across cnidarian lineages remain to be explored. Nevertheless, the present dataset offers a valuable resource for future functional and translational studies on sea anemone venom proteins.

## 4. Materials and Methods

### 4.1. Collection and Maintenance of Specimens

Individuals of wild *H. magnifica* were collected from offshore areas of Sanya City, located in the South China Sea, and then transported to the indoor aquarium at Hainan Medical University. The acclimation period lasted for two weeks, with a temperature of 25 °C, salinity of 33–35‰ in artificial seawater, and a 12 h light/dark cycle. For tissue collection, the tentacle and column tissues were excised and frozen in liquid nitrogen until use.

### 4.2. RNA Extraction, Illumina Sequencing and Transcriptome Assembly

Total RNA was extracted separately from the tentacle and column tissues of *H. magnifica* using a RNeasy mini kit (Qiagen, Germantown, MD, USA). After assessing RNA quality and quantity, RNA-sequencing libraries were prepared using the NEBNext Ultra RNA Library Prep Kit (NEB, Ipswich, MA, USA). The qualified library was sequenced on an Illumina Novaseq 6000 (Illumina, San Diego, CA, USA) high-throughput sequencing platform. The applied sequencing strategy is PE 150. Each sample contained at least 6 GB of sequencing data. Raw RNA-seq reads were subjected to quality filtering and adapter trimming using Fastp v1.0. Clean RNA-seq reads were subjected to de novo transcriptome assembly using Trinity v2.15.1 with the trimmomatic option enabled. Transcripts shorter than 200 bp were discarded to remove low-quality and fragmented sequences. The completeness of the assembled transcriptome was assessed using BUSCO v6.0.0 with the anthozoa_odb12 lineage dataset under transcriptome mode. Read alignment and transcript abundance estimation were performed using Trinity v2.15.1. Raw cleaned RNA-seq reads were aligned to the de novo assembled transcriptome with Bowtie2, and transcript and gene level abundances were quantified by RSEM. Gene level expected count matrix was built with Trinity script. Differential expression analysis was carried out using edgeR v4.4.2 package in R Bioconductor. Genes with *p* value < 0.05 were considered differentially expressed.

### 4.3. Gene Annotation and Functional Enrichment

For functional annotation, the encoded protein sequences from our transcriptome were compared with reported animal toxin sequences available from several public databases, including NR, UniProt, KEGG, KOG and GO. To identify putative toxin candidates, a reciprocal BLASTp search (E-value 1 × 10^−10^ and matching length > 60%, https://blast.ncbi.nlm.nih.gov/Blast.cgi, accessed on 20 September 2023) was performed against the transcriptome data using toxin genes from Tox-Prot animal venom database and the NCBI non-redundant protein sequences (nr) database. The signal peptides in protein sequences were predicted using the SignalP v4.0 server. The classification of proteins (amino acid number ≥ 80) and peptides (amino acid number < 80) was manually accomplished using BLAST searches based on known superfamilies in the UniProt database.

### 4.4. Venom Preparation, Protein Identification and Annotation for Proteomic Analysis

For the extraction of proteins, each sample was mixed with lyse buffer containing protease inhibitor cocktail, vortexed, and homogenized three times using a high-throughput tissue grinder. Then mixture was incubated at 4 °C for 30 min with vortexing every 10 min, then centrifuged for 20 min at 4 °C. The supernatant was collected and ultrafiltered using 10 kDa spin columns. For digestion, protein samples (100 µg) were transferred to a new tube and made up to 100 µL with 8 M urea and riethylammonium bicarbonate (TEAB) buffer. Reduction was carried out with 0.5 M Tris (2-carboxyethyl) phosphine at 37 °C for 60 min, followed by alkylation with 1 M iodoacetamide at room temperature for 40 min in the dark. Proteins were precipitated with five volumes of cold acetone at −20 °C overnight. After centrifugation (12,000× *g*, 4 °C, 20 min), the pellet was washed twice with pre-chilled 90% acetone, resuspended in 100 µL of 100 mM TEAB, and digested with trypsin at 37 °C overnight. The resulting peptides were desalted using Pierce C18 Spin Tips (Thermo Fisher Scientific, Waltham, MA, USA) and dried in a speed-vacuum concentrator.

Protein identification was performed by LC-MS/MS technology. In brief, the trypsinized samples were analyzed by an Easy-nanoLC 1200 system (Thermo Fisher Scientific, USA) with Q-Exactive Plus mass spectrometer (Thermo Fisher Scientific, USA). Chromatography was performed using a 25 cm analytical column with a linear gradient from 35% to 100% of buffer B (80% acetonitrile with 0.1% formic acid) over 47 min, followed by a 12 min hold at 100% buffer B. The mass spectrometer was operated in positive ion mode (*m*/*z* 350–1800, resolution 70,000) using full-scan MS followed by higher-energy collision dissociation (HCD) with a normalized collision energy of 28. Otherwise, the same data processing conditions and annotations were used as in the earlier study [10]. The mass spectrometry proteomics data have been deposited to the ProteomeXchange Consortium via the PRIDE [63] partner repository with the dataset identifier PXD081985 and PXD082090.

### 4.5. Bioinformatic Analysis

To identify sea anemone proteins, a manual BLAST search of the InterPro and UniProt database was performed, followed by analysis and validation of the cysteine frameworks of the protein toxins. The identified proteins were then assigned to known families in the BLAST and InterPro database. In the present study, a sequence containing more than 80 amino acids was defined as a protein. GO annotations, KEGG and KOG analyses were performed using the corresponding databases and visualized using the online tools available at (https://cnsknowall.com/#/HomePage, accessed on 10 March 2025). Phylogenetic analysis was performed using MEGA7.0.14 with the Neighbor-Joining method. Bootstrap support was assessed using 1000 pseudo-replicates. The phylogenetic tree was visualized and annotated within the online platform [64].

### 4.6. Alignment, Homology Modeling and Molecular Docking

Multiple alignments of some selected sequences were performed using Jalview 2.11.4.0 and TBtools under Clustal color scheme. The three-dimensional structures of the target proteins based on their mature region of amino acid sequences were predicted using AlphaFold v3.0. Molecular docking analysis between the proteins and targets was performed with Discovery Studio 2019. The docking scores of individual poses were calculated with the ZDOCK module. High-scoring complex poses were then refined using the RDOCK model. The pose with the lowest interaction energy was selected as the most favorable complex. The binding modes were retrieved and visualized with PyMOL 3.0. Interaction analysis was performed using the Analyze Protein–Protein Interactions tool and the data were visualized using a radar chart in Origin 2026.

## 5. Conclusions

The present work revealed, for the first time, a holistic overview of diverse proteins from *Heteractis magnifica* through the integrated transcriptomic and proteomic analysis. A total of 3573 protein sequences were identified, including 1835 from the column and 1925 from the tentacle, respectively. These sequences were assigned to hundreds of known families, which were further clustered into nine functional groups. We further examined the proteome of *H. magnifica*. After aligning the transcriptome against the proteome data, we recovered 339 proteins shared by the two datasets. Moreover, we selected several representative proteins from typical categories, including functional enzyme, functional protein, hemostatic and hemorrhagic, allergens and innate immunity, protease inhibitor and neurotoxin. Multiple alignments, homology modeling, and molecular docking were performed to screen for putative proteins with potentially high activity. Some low-abundance yet potentially important proteins were also identified, including insulin-like proteins, heat shock proteins, translin, Blot-5 and Ricin_B_lectin. Hence, this work provides a more in-depth understanding of the diverse proteins of *H. magnifica* and a novel information for the developing of potential candidates for therapeutic and biotechnological applications.

## Data Availability

The data that support the findings of this study are available from the corresponding authors upon reasonable request.

## Supplementary Materials

The following supporting information can be downloaded at https://www.mdpi.com/article/10.3390/md24080276/s1, Figure S1: Venn diagram showing the overlap of identified protein sequences between the tentacle and column transcriptomes; Figure S2: Venn diagram of putative proteins identified in the proteomes; Figure S3: GO analysis of the column proteome of *H. magnifica*; Figure S4: GO analysis of the tentacle proteome of *H. magnifica*; Figure S5: KEGG pathway annotation of the column proteome from *H. magnifica*; Figure S6: KEGG pathway annotation of the tentacle proteome from *H. magnifica*; Figure S7: KOG analysis of *H. magnifica* tentacle proteome; Figure S8: KOG analysis of *H. magnifica* column proteome; Figure S9: Docking result of reference proteins and optimal protein (PLA_2_); Figure S10: Docking result of reference proteins and optimal protein (Collagen); Figure S11: Docking result of reference proteins and optimal protein (EGF); Figure S12: Sequence alignment of representative proteins from the F5_F8_type_C family and known F5/8 type C domain-containing proteins; Figure S13: Sequence alignment of representative transcripts of FXa inhibition proteins Figure S14: Docking result of reference proteins and optimal protein (Ig); Figure S15: Docking result of reference proteins and optimal protein (Kunitz); Figure S16: Docking result of reference proteins and optimal protein (ShK); Table S1: Annotation of all protein sequences from tentacle of *H. magnifica*; Table S2: Annotation of all protein sequences from column of *H. magnifica*; Table S3: Annotation of protein sequences identified both in tentacle and column of *H. magnifica*; Table S4: The protein sequences expressed both in transcriptome and proteome; Table S5: Proteomics data of tentacle (peptides); Table S6: Proteomics data of tentacle (protein); Table S7: Proteomics data of column (peptides); Table S8: Proteomics data of column (protein).

## Abbreviations

Tissue Inhibitors of Metalloproteinases (TIMP);Whey Acidic Protein (WAP); Endomebrane Membrane Protein (EMP); ATP-binding cassette transporter (ABC_tran); Ligand-gated ion channel (Lig_chan); Secretory Carrier-Associated Membrane Protei (SCAMP); Dermatopontin (DERM); SOUL heme-binding protein (SOUL); Differential screening-selected gene Aberrant in Neuroblastoma (DAN); UDP-glucosyl transferase (UDPGT); Reverse Transcriptase (RVT); Lecithin-cholesterol acyltransferase (LCAT); Complement subcomponents C1r/C1s, Uegf, Bmp1 (CUB); Thrombospondin (TSP); Fibronectin (Fn); DOpamine β-MONooxygenase N-terminal domain (DOMON); Frizzled (Fz); Macroglobulin domain (MG); SEF/IL-17 receptor (IL-17R) domain (SEFIR); Bri/Chondromodulin/pro-SP-C (BRICHOS); Nidogen-like domain (NIDO); RNA Recognition Moti f (RRM_1); coagulation factor V and VIII type C (F5_F8_type_C); von Willebrand factor (VW); Epidermal Growth Factor-like domain (EGF); Histidine-Rich Glycoprotein (HRG); Immunoglobulin (Ig); Cysteine-rich secretory proteins, Antigen 5, and Pathogenesis-related 1 proteins (CAP);Leucine-Rich Repeat (LRR); Scavenger Receptor Cysteine-Rich (SRCR); Alpha-2-macroglobulin (A2M); Helix-turn-helix domain (HTH); Asp (Aspartic); Low-density lipoprotein receptor (Ldl_recept); Meprin/A5/mu (MAM); Domain of Unknown Function (DUF); Apextrin C-terminal domain (ApeC); Polycystin-1, Lipoxygenase, Alpha-Toxin (PLAT); Purple acid Phosphatase, N-terminal domain (Pur_ac_phosph_N); Receptor L domain (Recep_L_domain); Semaphorin (Sema); Tetratricopeptide repeat (TPR); Immunoglobulin V-set domain (V-set); protein kinase C substrate 80K-H (PRKCSH); Gamma-interferon-inducible lysosomal thiol reductase (GILT); Nuclear predominant protein NCU-G1 (NCU-G1); wingless Int-1 (Wnt); Fibroblast Growth Factor (FGF); Atrial Natriuretic Factor Receptor (ANF_receptor); Neurotransmitter-gated ion-channel (Neur_chan); Eph family receptor-interacting protein (Ephrin); Saposin A-type domain (SapA); Saposin B-type domain (SapB); A Disintegrin And Metalloprotease cysteine-rich (ADAM_CR); A Disintegrin And Metalloprotease (ADAM); tRNA N6-adenosine threonylcarbamoyltransferase family (TsaD); seven-transmembrane domain (7tm); Tumor Necrosis Factor Receptor cysteine-rich (TNFR_c6); Folate receptor (Folate_rec); Transmembrane protein (TMEM); Nicastrin small lobe (Ncstrn_small); Type VI Amidase Effector 4 (Tae4); Proteasome-activating nucleotidase 1 (PAN_1); Glycosyl hydrolases (Glyco_hydro); Protein tyrosine and serine/threonine kinase (His_Phos); Glycosyl transferase (Glyco_transf); Endonuclease/Exonuclease/phosphatase (Exo_endo_phos); 2OG-Fe(II) oxygenase (2OG-FeII_Oxy_3); Glycine-rich protein (Gly_rich).
